# The Natural Large Genomic Deletion Is Unrelated to the Increased Virulence of the Novel Genotype Fowl Adenovirus 4 Recently Emerged in China

**DOI:** 10.3390/v10090494

**Published:** 2018-09-13

**Authors:** Qing Pan, Jing Wang, Yulong Gao, Hongyu Cui, Changjun Liu, Xiaole Qi, Yanping Zhang, Yongqiang Wang, Xiaomei Wang

**Affiliations:** 1 State Key Laboratory of Veterinary Biotechnology, Harbin Veterinary Research Institute of Chinese Academy of Agricultural Sciences, Harbin 150001, China; panqing20050101@126.com (Q.P.); wangjing19951128@163.com (J.W.); ylg@hvri.ac.cn (Y.G.); cuihongyu@caas.cn (H.C.); liucj93711@hvri.ac.cn (C.L.); qxl@hvri.ac.cn (X.Q.); zhyp_77@hvri.ac.cn (Y.Z.); yqw@hvri.ac.cn (Y.W.); 2 Jiangsu Co-innovation Center for Prevention and Control of Important Animal Infectious Disease and Zoonoses, Yangzhou 225009, China

**Keywords:** novel FAdV-4, CRISPR/Cas9, natural deletion, genomic deletion, virulence

## Abstract

Since 2015, severe hydropericardium-hepatitis syndrome (HHS), caused by a highly pathogenic fowl adenovirus 4 (FAdV-4), emerged in China. In our previous study, the FAdV-4 has been identified as a novel genotype with a unique 1966-bp nucleotide deletion (1966Del) between open reading frame 42 and 43. In this study, the natural 1966Del was frequently identified among 17 clinical isolates and other reported Chinese clinical strains. To investigate the relationship between 1966Del and the increased virulence of the novel FAdV-4, a CRISPR/Cas9 operating platform for FAdV-4 was developed for the first time in this study. Based on this platform, a Re1966 strain was rescued, inserted the relative 1966Del sequence of a nonpathogenic strain KR5. In the pathogenicity study, the Re1966 strain retained high virulence for specific-pathogen-free chickens, similar to the parental wild-type HLJFAd15, although the survival time of chickens infected with Re1966 was much longer. Therefore, the natural 1966Del was identified as a non-essential site for the increased virulence of the emerged novel FAdV-4. Although further research on the virulence-determining region or point within the genome of the novel FAdV-4 is needed, the CRISPR/Cas9 operating platform for the novel FAdV-4 was developed and successfully applied to edit the genomic DNA for the first time, and it provides a novel powerful tool for both basic virology studies and vaccine vector development of FAdVs.

## 1. Introduction

Fowl adenoviruses (FAdVs) are classified in the family Adenoviridae, genus *Aviadenovirus*, and are classified into either five species (designated FAdV-A to FAdV-E) based on molecular criteria and restriction enzyme digestion patterns or twelve serotypes (designated FAdV-1 to 8a and -8b to 11) based on serum cross-neutralization tests [1]. The hexon is the major capsid protein of FAdV and contains the serotype-, and genotype-specific loop 1 region. According to the sequence of loop 1, serotype 1 is the only serotype within species A, serotype 5 is the only member within species B, serotypes 4 and 10 are members within species C, serotypes 2, 3, 9, and 11 are members within species D, and serotypes 6, 7, 8a, and 8b are members within species E, respectively.

FAdVs are non-enveloped, double-stranded DNA viruses [2] of poultry that are capable of causing hydropericardium-hepatitis syndrome (HHS), inclusion body hepatitis (IBH), or gizzard erosion (GE), resulting in significant economic losses to the poultry industry all over the world [3,4,5]. Almost all of the pathogenic FAdVs can induce IBH with or without mortality [6,7,8,9], whereas fowl adenovirus serotype 4 (FAdV-4) plays a primary role in the etiology of HHS, with mortality varying from 10 to 100%. HHS, associated with FAdV-4 infection, was first reported in Pakistan in 1987 [10], and subsequent outbreaks have been reported in Chile [11], Mexico [12], Korea [13], and India [14]. Recently, HHS associated with a novel FAdV-4 infection emerged in China [15,16].

In our previous studies, two pathogenic FAdV-4 strains were separately isolated from layers and ducks. The complete genomes were sequenced and aligned (GenBank Nos. KU991797 and KX538980) [17,18]. A significant natural large genomic deletion (1966 bp) was presented between open reading frames (ORF) 42 and 43 compared with the non-pathogenic strains ON1 (GenBank no. GU188428) and KR5 (GenBank no. HE608152), and both of the pathogenic strains were identified as a novel genotype FAdV-4 [16]. The lipase, which was encoded by the deleted gene, has been identified to be a virulence factor for Marek’s disease virus (MDV) and relative to cell entry for hepatitis C virus (HCV). These findings raised the question of whether the unique natural deletion in the novel genotype FAdV-4 is related to the adenovirus’ virulence and pathogenicity. To investigate the function of the natural deletion in the novel genotype FAdV-4, a rapid and efficient genetic modification tool for FAdV-4 DNA engineering is urgently needed.

DNA viruses with large genomes, such as adenoviruses, are difficult to manipulate. Traditional methods, such as homologous recombination (HR) or bacterial artificial chromosome (BAC), have been used to edit FAdV-1 [19] FAdV-4 [20], and FAdV-9 [21]. However, some disadvantages limit their application: HR is very time consuming because of its low efficiency, and BAC mutagenesis is only available based on a produced, useful BAC, which is difficult to obtain for some DNA viruses. Clustered regularly interspaced short palindromic repeats/CRISPR-associated protein 9 (CRISPR/Cas9) has emerged as a novel powerful genetic modification tool for DNA engineering in diverse organisms, and it has advantages over HR and BAC methods [22]. CRISPR/Cas9 operates by introducing double-stranded breaks at targeted DNA sites, and these breaks subsequently stimulate cell self-repair pathways within nonhomologous end joining (NHEJ) and homology-directed repair (HDR). Gene knockin or knockout of viral DNA can also be achieved by employing both of these repair pathways. CRISPR/Cas9 is easily applied because it requires only the design of effective single-guide RNA (sgRNA), and it has been used to edit human adenovirus (HAdV) [23], herpes simplex virus 1 (HSV-1) [22], hepatitis B virus (HBV) [24], Epstein-Barr virus [25], pseudorabies virus (PRV) [26], and herpesvirus of turkeys (HVT) [27]. Although CRISPR/Cas9 has been used to edit HAdVs, most of the studies involved have focused on the delivery vector function for gene therapy or vaccine construction rather than on pathogenesis. Furthermore, CRISPR/Cas9 has not previously been used to edit FAdVs, whose genomes (~45 kb) are approximately 10 kb larger than human adenovirus genomes (~35 kb) [28,29].

In this study, the natural large genomic deletion 1966Del was confirmed in all of 17 clinical isolates from chickens and ducks with severe HHS. To reveal the function of the natural deletion, CRISPR/Cas9 was used to manipulate fowl adenovirus for the first time, and a 1966Del knockin strain (Re1966) was subsequently rescued. Furthermore, the pathogenicity of rescued Re1966 was evaluated in specific-pathogen-free (SPF) chickens in comparison with the parental virus to identify the natural deletion, which is frequently present in highly virulent strains, as either a virulence-related factor or a non-essential replication site.

## 2. Materials and Methods

### 2.1. Viruses and Cells

HLJFAd15 (GenBank no. KU991797) and HLJDAd15 (GenBank no. KX538980) were isolated from layers and ducks, respectively, in our previous studies and shown to be highly virulent strains [17,18]. The remaining 15 clinical isolates were all from chickens with severe HHS from different provinces in China (Table 1). Chicken liver hepatocellular carcinoma cell line (LMH cells) were cultured [30] in Dulbecco’s modified Eagle’s medium (DMEM) (Thermo Fisher Scientific, Waltham, MA, USA) supplemented with 15% fetal bovine serum (HyClone, South Logan, UT, USA), 100 IU/mL penicillin and 100 μg/mL streptomycin.

### 2.2. Total DNA Extraction and Genome Sequencing

Total DNA was extracted from liver samples of chickens with severe HHS using a DNeasy Tissue Kit (Qiagen, Hilden, Germany) according to the manufacturer’s instructions and used as a template for PCR amplification. The L1 region of the hexon gene was sequenced to screen FAdV-4 using the following FAdV serotype-specific primers [31]: FAdF (5′-AACTTCGACCCCATGTCGCGTCAGG-3′) and FAdR (5′-TGGCGAAAGGCGTACGGAAGTAAGC-3′). Then, a phylogenetic tree based on the hexon L1 region was constructed using MEGA 6.0 software (http://www.megasoftware.net/) by the maximum-likelihood method (1000 bootstrap replicates). Partial sequences from pVIII to GAM-1 of the selected FAdV-4 were subsequently detected by PCR and manually assembled using the Seqman program of the DNAstar software package (version 5.01, Madison, WI, USA).

### 2.3. Purified Viral DNA Preparation

Cell cultures were collected from the infected cells 72 h post infection (hpi) with visible CPE. After three cycles of freezing and thawing, the mixture was centrifuged at 5000× *g* for 30 min to remove cellular debris and subsequently centrifuged at 18,000× *g* for 2 h to pellet virions. The virus pellet was further centrifuged in cesium chloride (CsCl) overnight to collect the virus enrichment layer. Finally, the virus was resuspended in PBS and stored at −80 °C until use. The DNA was extracted from the purified virions for transfection or stored at −20 °C until use.

### 2.4. Donor Plasmid Construction

The left 1000-bp recombinant arm (ReA) and right 1000 bp arm (ReB) adjacent to the 1966Del site were amplified by PCR from the genomic DNA of HLJFAd15 and subsequently cloned into plasmid pUC19 (GenBank no. X02514) at the EcoR I and Kpn I enzyme sites separately to obtain pUC19AB. The enhanced green fluorescent protein (EGFP) cassette within a CMV promoter from pEGFP-N1 (Clontech Laboratories, Mountain View, CA, USA) and the relative 1966Del sequence of a non-pathogenic strain KR5 were synthesized from a commercial company (Genewiz, Nanjing, China) were cloned into pUC19AB between ReA and ReB by homologous recombination to obtain a non-gap connection ReA-EGFP/1966Del-ReB fragments contained in pUC19AB-EGFP or pUC19AB-1966Del.

### 2.5. Generation of CRISPR/Cas9 sgRNA Plasmids

All sgRNAs involved in this study (Table 2) were designed using the online CRISPR Design Tool (https://wwws.blueheronbio.com/external/tools/gRNASrc.jsp) to target the site where the natural 1966Del occurred. Plasmid pX330, a human codon-optimized SpCas9 and the chimeric guide RNA expression vector, was acquired as an indirect gift from Feng Zhang [26,32]. The pX330 plasmid was digested using Bbs I (ThermoScientific, Waltham, MA, USA), and then the CRISPR/Cas9 constructs were constructed. All of the constructs in this study were verified by sequencing (Jilin Comate Bioscience Co., Ltd., Changchun, China).

### 2.6. Cotransfection and Virus Rescue

The cotransfection procedure for the HLJFAd15 genome, ReA-EGFP/1966Del-ReB DNA fragments, and different CRISPR/Cas9 sgRNAs was performed as previously reported [27] with slight modification. Briefly, 1.5 μg of the purified HLJFAd15 genome, 1.5 μg of purified PCR product of ReA-EGFP/1966Del-ReB fragments, and 0.5 μg of pX330-sgRNA were added into each individual well of a 6-well plate using 10.5 μL of X-tremeGene HP DNA Transfection Reagent according to the manufacturer’s instructions (Roche, Basel, Switzerland). Seventy-two hours post transfection (hpi), the cells were collected and subjected to three cycles of freezing and thawing. The recombinant virus containing the EGFP expressing cassette (ReEGFP) or relative natural deleted 1966 bp DNA of KR5 stain (Re1966) was purified with plaque purification in LMH cells overlaid with 1% low-melting-point agarose (Lonza, Rockland, ME, USA) and DMEM with 2% FBS. For the screening of high-efficiency sgRNAs for the cleavage of the 1966Del site, six sgRNAs were detected by calculating the percentage of EGFP-positive plaques among all of the virus plaques. The GFP-positive plaques were counted under an inverted fluorescence microscope, and the negative plaques were stained with a crystal violet assay. Finally, the recombination ratios were calculated. A wild-type virus was also rescued by transfection with the purified viral DNA.

### 2.7. Identification the Stability and Growth Properties of the Rescued Viruses

All 3 of the rescued strains, wild type, ReEGFP, and Re1966, were propagated in LMH cells for 10 passages. The stability of the insertion of the EGFP cassette or 1966Del was separately detected by fluorescence observation or PCR assay using primers located at the recombinant arms: Ad29F (5′-GTTCAGATCCAATATCGCCA-3′) and Ad29R (5′-GTTAGCAAGTGGAAAGTTCCA-3′). To assess the replication stability of the rescued viruses, the cloned viruses were inoculated into monolayer LMH cells at a multiplicity of infection (MOI) of 0.01, and viruses were harvested at 12, 24, 36, 48, 60, 72, 84, and 96 hpi. Then, the infected cells were frozen and thawed three times, and the cell supernatants were serially diluted 10-fold from 10^−3^ to 10^−8^ and applied to a plaque assay. The plaque forming units (PFU) were determined at 7 dpi. All data are shown as the means ± SD of three independent experiments. Furthermore, the third-passage viral cultures of the rescued viruses were harvested and subjected to genomic sequencing following a previously reported method [18]. 

### 2.8. Pathogenicity Analyses of the Rescued Viruses

Sixty 28-day-old specific-pathogen-free (SPF) chickens were purchased from the Experimental Animal Centre of Harbin Veterinary Research Institute (HVRI, Harbin, China) of the Chinese Academy of Agricultural Sciences (CAAS, Beijing, China). The chickens were randomly divided into three groups with 20 birds per group and individually housed in negative-pressure isolators. Each chicken was inoculated with 0.5 mL of virus containing 5 × 10^5^ PFU HLJFAd15 or Re1966 via intramuscular injection and monitored daily for 10 days. The chickens in the control group were mock inoculated with the same dose of LMH cell culture supernatant.

In the necropsy examination, FAdV infection-related clinical signs, hydropericardium syndrome, hepatitis, and gastric injury were observed. Tissue samples, including heart, liver, spleen, lung, kidney, thymus, bursa, and proventriculus samples, were collected from the dead and control chickens and subjected to real-time PCR assay [33] for the detection of the virus distribution in different tissues. Pathologically changed tissues (liver, kidney, and proventriculus) were further submitted to a histopathology assay to confirm the pathogenesis of each of the rescued viruses. 

### 2.9. Ethics Statement

The animal experiments with chickens (License no. SYXK 2017-009) were approved by the Ethical and Animal Welfare Committee of Heilongjiang Province, China (License No. SQ20150508) and performed in accordance with the ‘Guidelines for Experimental Animals’ of the Ministry of Science and Technology (Beijing, China). All SPF chickens were cared for in accordance with humane procedures.

### 2.10. Statistical Analysis

Data were expressed as the means ± SD and analyzed using ANOVA as implemented in SPSS version 19.0 (IBM, Armonk, NY, USA). Differences were considered statistically significant at * *p* < 0.05.

## 3. Results

### 3.1. The Natural Large Genomic Deletion (1966Del) Specifically Characterized in the Novel Chinese FAdV-4 Strains

**Figure 1:**
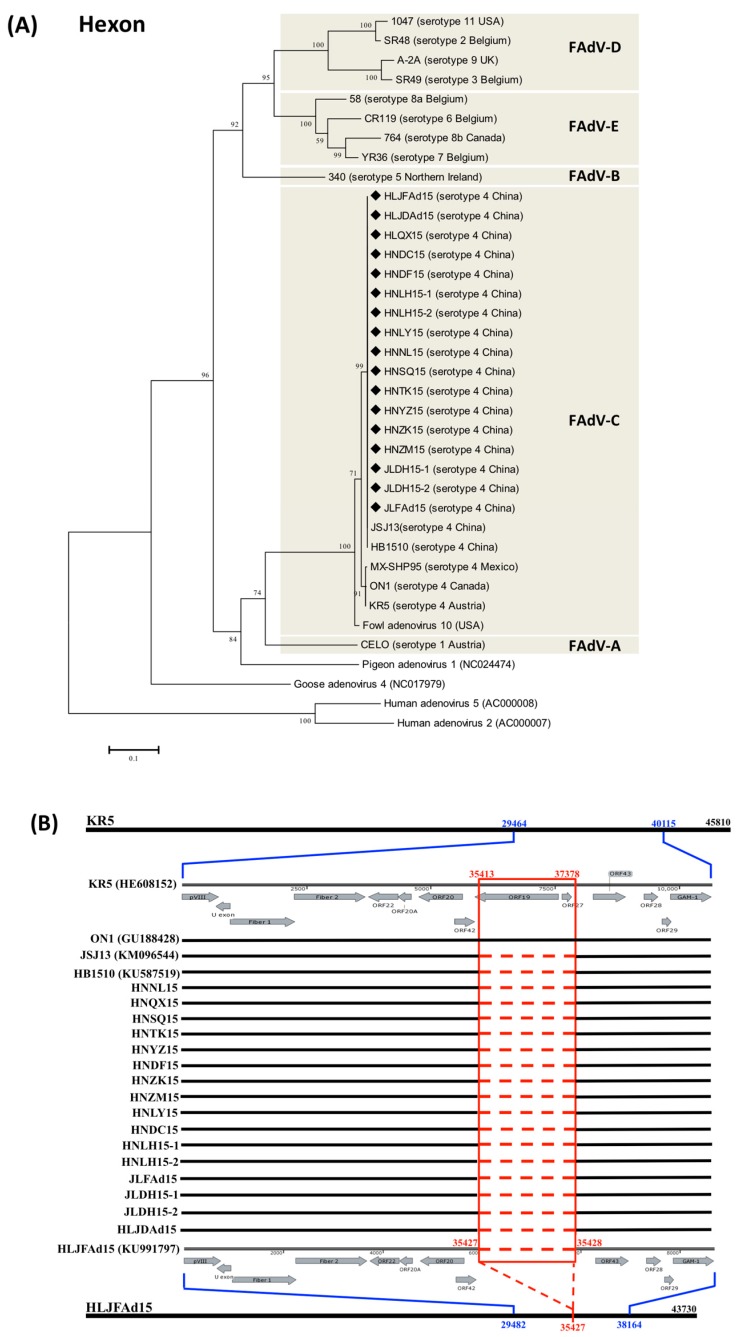
Investigation of the prevalence of the natural large genomic deletion in the novel genotype FAdV-4 that recently emerged in China. (**A**) Phylogenetic analysis of FAdV clinical isolates based on the sequence of hexon-L1. Seventeen clinical isolates were characterized as FAdV-4: HLJFAd15 (KU991797) and HLJDAd15 (KX538980) were isolated from chickens and ducks from Heilongjiang Province; JLFAd15, JLDH-1, JLDH-2 were isolated from chickens from Jilin Province; and HNNL15, HNQX15, HNSQ15, HNTK15, HNYZ15, HNDF15, HNZK15, HNZM15, HNLY15, HNDC15, HNLH15-1, and HNLH15-2 were isolated from chickens from Henan Province. (**B**) Partial genome organization from pVIII to GAM-1 of FAdV-4 isolates. The natural 1966 bp deletion mutant, including the lipase reading frame, presented between ORF42 and ORF43 in 17 clinical, highly pathogenic isolates and another two reported Chinese clinical strains, JSJ13 (KU991797) and HB1510 (KU587519), but did not appear in KR5 (HE608152) and ON1 (GU188428) isolated outside of China.

Based on the serotype-specific sequence of the L1 region of hexon, 17 novel genotype FAdV-4 strains were isolated and identified (Figure 1A) from chickens with severe HHS from different farms in three different provinces (Heilongjiang, Jilin, and Henan) of China. The similarity of the hexon sequence between the novel genotype FAdV-4 and each of the other FAdV-4 strains and other species of FAdVs was varied from 98.6–100% and 50.1–76.2%, respectively. The complete genome sequences of HLJFAd15 from layers and HLJDAd15 from ducks (identified as the novel genotype FAdV-4) were submitted to GenBank during our previous study [17,18]. The large genomic deletion 1966Del, located between ORF42 and 43, was sequenced and its absence confirmed in all of the other 15 clinical strains from chickens with emerged HHS, but did not appear in KR5 (HE608152) and ON1 (GU188428) isolated outside of China. (Figure 1B). 

### 3.2. Development of the CRISPR/Cas9 Platform to Manipulate FAdV-4

**Figure 2:**
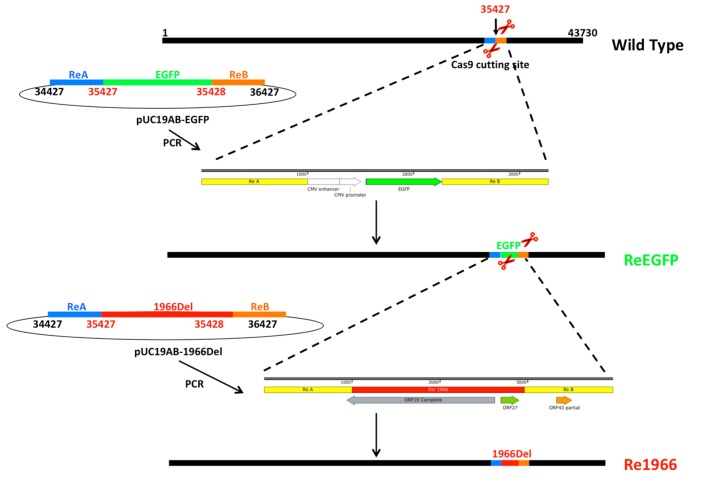
Strategy of the CRISPR/Cas9 platform for the novel genotype FAdV-4. Co-transfection with purified HLJFAd15 genome DNA, ReA-EGFP/1966Del-ReB sequences containing the recombinant arms, and pX330-sgRNA into LMH cells were used to rescue recombinant viruses. The ReEGFP strain was rescued first for screening of the sgRNA with the highest efficiency in targeting the site where the deletion occurs. Then, the Re1966 strain containing the 1966Del sequence of KR5 (HE608152) was rescued.

**Figure 3:**
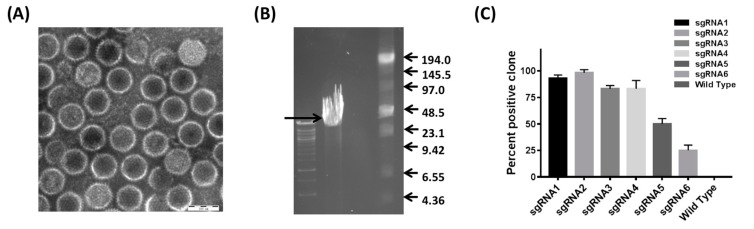
The cleavage ratio of variant sgRNAs targeting the site of the deletion. (**A**) Purified virus particles obtained by CsCl2 gradient centrifugation. Scale bar= 100 nm. (**B**) The viral genome DNA was of high quality and purity, with a molecular size of approximately 43 kb. (**C**) Cleavage ratios were detected by calculating the percentage of GFP-positive clones among the total virus clones. sgRNA1-4 showed efficient cleavage effects for editing FAdV-4 DNA (>80%). The sgRNA2 showed a cleavage ratio of 98.3 ± 1.6% and was used for the subsequent targeting.

To establish the CRISPR/Cas9 platform (Figure 2), purified high-quality virons were obtained via CsCl gradient centrifugation (Figure 3A). High-purity, complete DNA, with a molecular size of approximately 43 kb, was subsequently extracted from the purified virons (Figure 3B). For the screening of high-efficiency sgRNAs for the cleavage of the 1966Del site, six sgRNAs were detected by calculating the percentage of EGFP-positive plaques among all of the virus plaques. The results (Figure 3C) showed that sgRNA1-4 showed efficient cleavage effects for editing FAdV-4 DNA (>80%). In particular, sgRNA-2 yielded a cleavage ratio of 98.3 ± 1.6% and was used for the subsequent targeting.

### 3.3. Stability and Growth Properties of the Rescued Viruses

**Figure 4:**
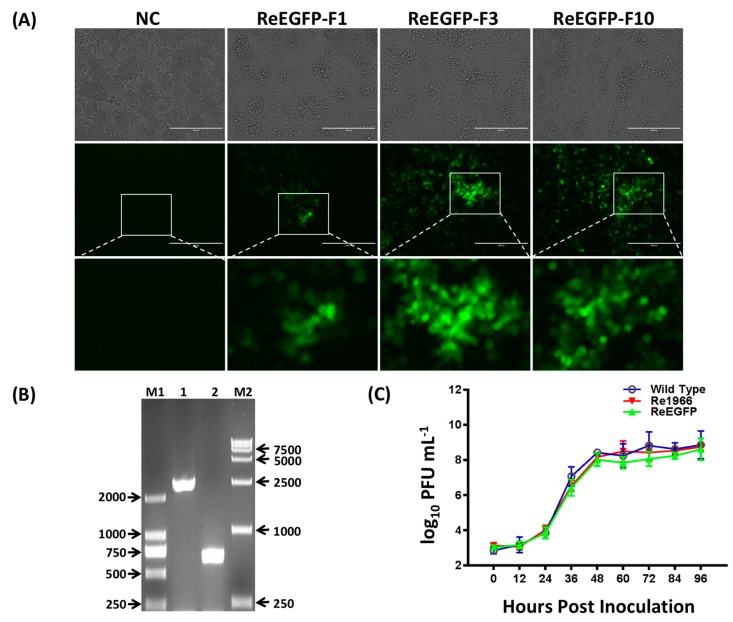
Stability and growth properties of the rescued viruses. Recombinant viruses ReEGFP/Re1966 with insertion of a EGFP expression cassette or the natural deletion 1966 bp sequence were both rescued by the CRISPR/Cas9 platform. (**A**) The ReEGFP strain was able to continuously express GFP for at least 10 passages. Scale bar= 200 μm. (**B**) The 1966Del insertion was also detected in the rescued Re1966 virus by PCR. A 658 bp sequence was amplified from the wild-type strain (Lane 2), and a 2624 bp sequence with the 1966Del insertion was detected in the Re1966 strain (Lane 1). (**C**) There was no significant difference in any of the growth properties in vitro among the 3 rescued viruses: ReEGFP, Re1966, and wild-type.

Recombinant viruses of ReEGFP and Re1966 with insertion of the EGFP expression cassette or the natural deletion 1966 bp sequence were both rescued by the CRISPR/Cas9 platform. The EGFP was able to continuously express in the rescued ReEGFP virus for at least 10 passages (Figure 4A). The insertion of 1966Del genomic part was also detected in the rescued Re1966 virus by PCR and stably propagated for 10 passages (Figure 4B). A 658 sequence was amplified by PCR from the wild-type strain, and a 2624 bp sequence with the insertion of 1966 bp was detected in the Re1966 strain. There was no significant difference in any of the in vitro growth properties among the rescued ReEGFP, Re1966, and wild-type strains (Figure 4C).

### 3.4. Pathogenicity of Re1966 and the Wild-Type Strain HLJFAd15 in SPF Chickens

**Figure 5:**
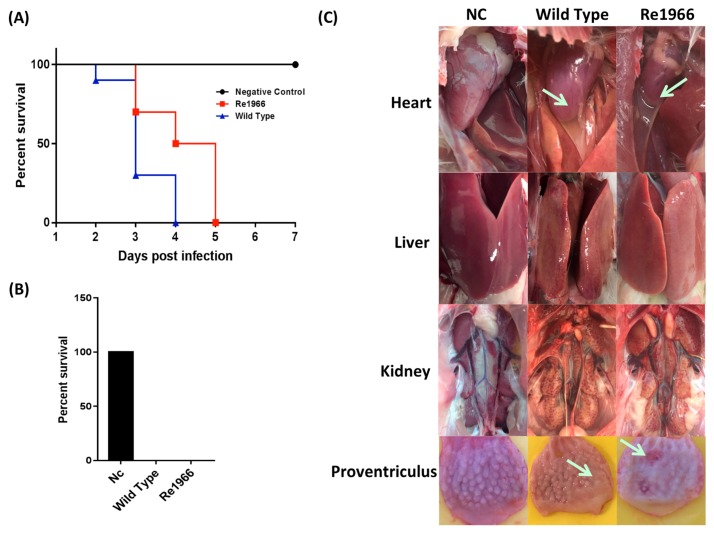
Analyses of the pathogenicity of the rescued Re1966 for SPF chickens. All of the SPF chickens were observed for 10 dpi. The chickens in the wild type and Re1966 strain groups died within 2 to 5 dpi (**A**), and the mortalities of both the groups were 100% (**B**). The survival time of chickens infected with Re1966 was greatly prolonged relative to that of the birds infected with the wild-type strain. (**C**) In the necropsy examination, all of the dead chickens in both infected groups showed severe hydropericardium syndrome, hepatitis (swollen and friable liver), proventriculus erosion, and kidney enlargement kidney, and there was no significant difference in these features between the two infected groups. All of the SPF chickens in the negative control group remained alive and did not show any clinical signs.

All of the SPF chickens were observed for 10 days post infection. The chickens in the wild-type and Re1966 strain groups died within two to five days (Figure 5A). The mortalities of both groups were 100% (Figure 5B), although the survival time of chickens infected with Re1966 was greatly prolonged relative to that of the birds infected with the wild-type strain. In the necropsy examination, all of the dead chickens in both infected groups showed severe hydropericardium syndrome, hepatitis (swollen and friable liver), proventriculus erosion, and kidney enlargement, and there was no significant difference in these features between the two infected groups (Figure 5C). All of the SPF chickens in the negative control group remained alive and did not show any clinical signs over the 10 days post inoculation with mock LMH cell culture supernatant.

### 3.5. Histopathology Assay and Virus Distribution

**Figure 6:**
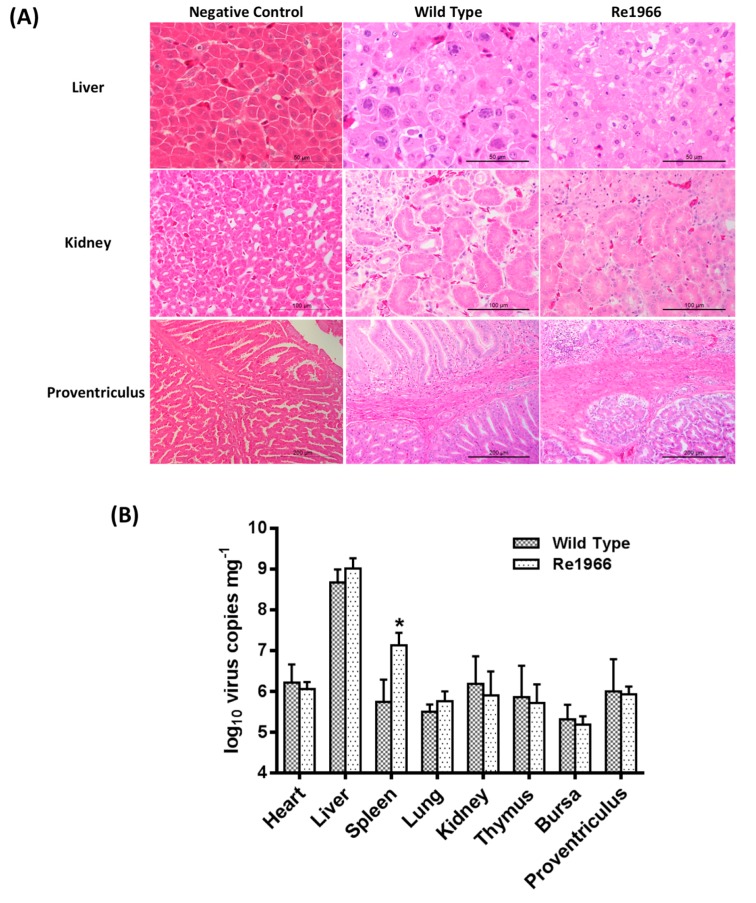
Histopathology and virus loads in tissues of chickens inoculated with the rescued viruses. (**A**) Histopathology assays of the major targeted tissues by FAdV-4 infection. Generally, there was no significant histopathology presented in tissues of chickens in the control group. However, degeneration, vacuolar necrosis, and basophilic inclusion bodies presented in liver cells, large numbers of vacuolar necrosis showed in kidney cells, and partial lymphocyte necrosis appeared in the lamina propria of the proventriculus mucous membrane in the chickens inoculated with viruses. There was no significant difference in these features between chickens inoculated with the wild-type strain and those inoculated with the Re1966 strain. (**B**) Virus loads in different tissues of chickens inoculated with FAdV-4. The Re1966 strain showed the same distribution and replication kinetics as the wild-type strain in the different tissues of SPF chickens except for a higher number of virus copies in spleen (* *p* < 0.05).

To investigate the different pathogenicities of the wild-type and Re1966 strains, sections of the liver, kidney, and proventriculus were stained with hematoxylin and eosin (HE) and examined using light microscopy (Figure 6A). Typical inclusion bodies were observed in the liver cells of chickens in both infected groups. Degeneration of partial renal tubular epithelial cells and congestion was observed in the kidneys. Partial lymphocyte necrosis appeared in the propria of the proventriculus mucous membrane. However, no significant differences in these features were observed between the infected groups. The distributions of the wild-type and Re1966 strains in different tissues were investigated (Figure 6B). The Re1966 strain showed the same distribution and replication kinetics as the wild-type strain in the different tissues of SPF chickens except for a higher number of virus copies in spleen (*p* < 0.05). None of the SPF chickens in the negative control group showed any histopathology changes, and virus DNA was not detected by real-time PCR in this group throughout the experiment.

## 4. Discussion

In June 2015, a natural outbreak of hydropericardium-hepatitis syndrome (HHS), with a mortality rate of 30–90% in chickens, occurred in most of the high-intensity poultry farming provinces in China, and the disease rapidly spread to different areas of the country [14,34]. In our previous study, HHS appeared not only in chickens [17,33], but also in ducks, with a mortality of 15% [18]. A highly virulent FAdV-4, identified as a novel genotype [16], was found to be the responsible pathogenic factor. Two novel genotype FAdV-4 strains, isolated from layers and ducks, respectively, were identified and their complete genomes sequenced in our previous studies [17,18]. In examining the partial sequences of 15 isolates from this study and some other reported Chinese strains [13,16], a notable large genomic deletion (1966 bp) was identified and found to be identical to the novel genotype FAdV-4.

To investigate the relationship between the natural 1966Del and the enhanced virulence of the novel genotype FAdV-4 that emerged in China, a DNA engineering platform was needed to manipulate the large genome of FAdV-4. Although a homologous recombination method based on a cosmid system has been developed for FAdV-1 (species A) and FAdV-9 (species D) [19,21], the low efficiency and establishment of the BAC limits its application. The CRISPR/Cas9 platform has been widely used for the construction of vaccines and gene therapy based on the vector function of the nonpathogenic human adenovirus [23]. For the first time, the CRISPR/Cas9 tool was applied in the present study to manipulate FAdVs, whose genomes are approximately 10 kb larger and more difficult to manipulate than human adenovirus genomes [28,29]. The ReEGFP strain was rescued for screening efficient sgRNAs for targeting the site of the natural deletion, and the 1966Del knockin strain Re1966 was subsequently rescued based on the efficiency of sgRNA-2, which had an approximately 100% cleavage ratio. All of the materials used in this study, including the viral genome DNA, purified PCR product of ReA-Insertion-ReB, and pX330-sgRNA expressing Cas9, are easily prepared. The high rescue efficiency greatly reduced the time required for screening the positive clones. Thus, the easily operated and high-efficiency CRISPR/Cas9 platform for FAdV-4 developed in this study can be considered a powerful tool for the screening of virulent factors and pathogenesis studies of all members of FAdVs.

Natural genomic deletions, such as the one that appeared in the emerged viruses in this study, usually play important roles in virus evolution and virulence changes. A natural deletion in the NS gene of H5N1 swine influenza virus induced attenuation in chickens [35]. A 205-nucleotide deletion in the 3′-UTR of avian leucosis virus subgroup J (ALV-J) enhanced the virus’ pathogenicity [36]. However, the 30-amino-acid deletion in the Nsp2 gene of highly pathogenic porcine reproductive and respiratory syndrome virus (PRRSV) was found to be unrelated to the virus’ virulence [37]. To investigate the relationship between the natural deletion and the enhanced virulence of the novel genotype FAdV-4, the Re1966 strain containing the natural deletion 1966Del of a nonpathogenic strain KR5 was rescued based on the CRISPR/Cas9 platform developed in this study. Unfortunately, the results showed no significant difference between the rescued Re1966 and the wild-type HLJFAd15 strains in either the growth properties in vitro or the pathogenicity in vivo. The lipase reading frame, contained in the natural genomic deletion, is conserved in certain avian adenoviruses, and homologues are found in members of the Marek’s disease virus (MDV) and identified as virulence factor of MDV [38,39]. However, there are two homologues (ORF19 in the negative strand and ORF19A in the positive strand) of lipase that appear in FAdV-4 and FAdV-10 but not other serotypes of FAdVs [40]. The high protein-coding potential of ORF19A, providing compensatory expression of lipase in the novel genotype FAdV-4 without ORF19 (low protein-coding potential) [41], might be the reason why the lipase reading frame was related to the virulence of MDV but not FAdV-4. Furthermore, strain SHP95 [12], isolated from Mexico, contains a truncated ORF19, but retains high pathogenicity, which is consistent with the lack of relationship between the natural deletion of ORF19 and the virulence of the novel FAdV-4. Further research on the virulence-determining region or point within the genome of the novel genotype FAdV-4 using the efficient CRISPR/Cas9 platform developed in this study is essential.

## 5. Conclusions

A natural large genomic deletion (1966Del), located between ORF42 and 43, was confirmed in all of the 17 novel genotype clinical FAdV-4 strains from chickens with HHS. A CRISPR/Cas9 operating platform was successfully developed and applied to edit the genomic DNA of FAdVs for the first time in this study, which provides a novel powerful tool for both basic virology studies and vaccine vector development of FAdVs. Based on the developed CRISPR/Cas9 system, the natural large genomic deletion (1966Del) was knocked-in and further identified as a non-essential mutant for the pathogen’s increased virulence of the novel emerged FAdV-4. Further research on the virulence-determining region or point within the genome of the novel genotype FAdV-4 is essential.

## Figures and Tables

**Table 1:** Information on the 17 clinical strains used in this study.

No.	Name	Location	Host	Age	Clinical Signs ^a^	Mortality
1	HLJFAd15	Zhaodong, Heilongjiang	Layers	180	HHS, IBH	>50%
2	HLJDAd15	Shuangcherng, Heilongjiang	Ducks	45	IBH	15%
3	JLFAd15	Jiutai, Jilin	Layers	72	HHS, IBH	18%
4	JLDH-1	Dehui, Jilin	Broiler breeders	160	HHS, IBH	15%
5	JLDH-2	Dehui, Jilin	Broiler breeders	160	HHS, IBH	15%
6	HNNL15	Ningling, Henan	Layers	46	HHS, IBH	61%
7	HNQX15	Qixian, Henan	Layers	45	HHS, IBH	43%
8	HNSQ15	Shenqiu, Henan	Layers	90	HHS, IBH	32%
9	HNTK15	Taikang, Henan	Layers	55	HHS, IBH	62%
10	HNYZ15	Yuzhou, Henan	Layers	52	HHS, IBH	42%
11	HNDF15	Dengfeng, Henan	Layers	46	HHS, IBH	46%
12	HNZK15	Zhoukou, Henan	Layers	34	HHS, IBH	52%
13	HNZM15	Zhongmu, Henan	Layers	64	HHS, IBH	36%
14	HNLY15	Linying, Henan	Layers	55	HHS, IBH	56%
15	HNDC15	Dancheng, Henan	Layers	63	HHS, IBH	48%
16	HNLH15-1	Luohe, Henan	Layers	44	HHS, IBH	57%
17	HNLH15-2	Luohe, Henan	Layers	52	HHS, IBH	41%

^a^ HPS, hydropericardium syndrome. IBH, inclusion body hepatitis.

**Table 2:** Sequences of sgRNA oligonucleotides.

Name	Sequences (5′-3′)	Name	Sequences (5′-3′)
sgRNA-1	CACCGATTAGGTCGCGCATTCCGAT	sgRNA-4	CACCGTATATGGCGGATGTTCGGAT
	AAACATCGGAATGCGCGACCTAATC		AAACATCCGAACATCCGCCATATAC
sgRNA-2	CACCGCGGATCGGAGTTTATTCGCC	sgRNA-5	CACCGCTCAGAGGCTCCTTCTCGAG
	AAACGGCGAATAAACTCCGATCCGC		AAACCTCGAGAAGGAGCCTCTGAGC
sgRNA-3	CACCGAATGCGCGACCTAATATCAC	sgRNA-6	CACCGTTACGAAAGCTGTGTTTATA
	AAACGTGATATTAGGTCGCGCATTC		AAACTATAAACACAGCTTTCGTAAC
